# Production of polyhydroxyalkanoate (PHA) biopolymer from crop residue using bacteria as an alternative to plastics: a review

**DOI:** 10.1039/d4ra08505a

**Published:** 2025-04-15

**Authors:** Aakriti Chouhan, Archana Tiwari

**Affiliations:** a School of Biomolecular Engineering & Biotechnology, Rajiv Gandhi Proudyogiki Vishwavidhyalaya (University of Technology of Madhya Pradesh), Accredited with Grade “A” By NAAC Airport Road Bhopal-462033 India chouhanaakriti@gmail.com archanargpv@gmail.com

## Abstract

Growing environmental concerns and the pressing need to combat plastic pollution have led to extensive research on sustainable alternatives to traditional plastics. Human blood sample analysis discovered microplastics which has caused health concerns regarding their influence on proper functioning of the human body. The compound polyhydroxyalkanoate (PHA) has gained popularity due to its comparable structure with synthetic polymers like polypropylene because it belongs to the category of biodegradable alternatives. Different PHA molecules have distinct properties because of their composition of monomers and production parameters. The current market offers an array of biopolymers but they do not satisfy industrial requirements regarding thermostability. The industrial heat-stability of materials comes from green biomass-derived polyethylene and extrudable cellulose biopolymers. The research analyses PHAs' suitability as synthetic plastic substitutes and addresses barriers to their industrial production and proposes modifications to improve performance. It underscores the importance of harnessing crop residue streams to produce valuable biopolymers, promoting resource efficiency and mitigating the environmental impact of plastic waste. This work aligns with the UN's sustainability goals, including SDG 3 good health, SDG 11 sustainable cities, SDG 12 responsible consumption, SDG 13 climate action, and SDG 14 sea and ocean protection.

## Abstract 2

Production of polyhydroxyalkanoate (PHA) biopolymer from crop residue using microbial strain.

## Graphical abstract

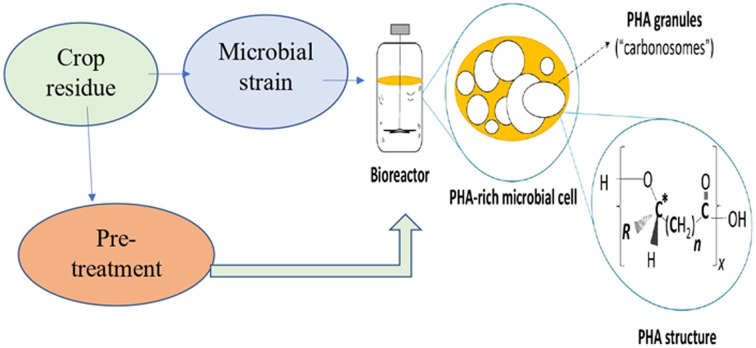

## 1. Introduction

**Fig. 1:**
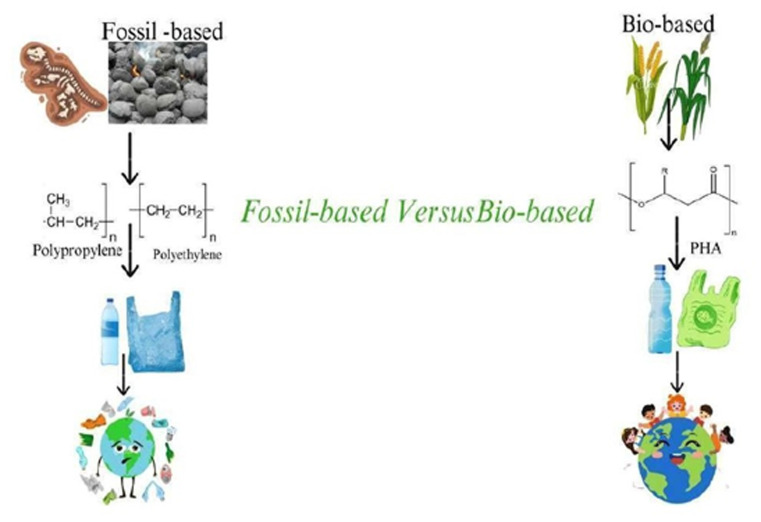
Synthetic polymers and biopolymers. (Synthetic polymers derived from fossil-based compounds are environmentally unfriendly such as pollypropylene, and polyethylene. Biopolymers that are derived from living organisms or bio-based resources are environmentally friendly such as polyhydroxyalkanoates).

Every year, millions of tons of plastic are produced and discarded after a single use, contributing to environmental pollution.^1^ Recently research in the Netherlands revealed microplastic pollution in human blood for the first time. Analyzing blood samples from 22 donors, scientists discovered that 17 individuals had been affected by microplastics—tiny fragments of synthetic polymers measuring less than 5 mm. These microplastics can enter the animal and human food web, posing potential risks to health as they travel throughout the body *via* the bloodstream. In the current era of technological advancements, almost every product contains some form of polymer. The increasing demand for polymers and the products they constitute has been traditionally met by synthetic polymers, commonly known as plastics. However, the production of these plastics, derived from fossil-based compounds, is not only expensive but also environmentally unfriendly.^2^ A potential solution to this environmental challenge is the use of biopolymers, which are derived from living organisms or bio-based resources as shown in Fig. 1.^3,4^ Replacing widely used substances with biopolymers is no easy task, as any substitute must closely resemble the characteristics of the original product for consumer adaptation.^5^ Fortuitously, a unique class of biopolymers known as polyhydroxyalkanoates (PHAs) exhibits extraordinary similarities to well-established synthetic polymers like Polypropylene in mechanical strength and crystallinity.^6,7^ PHAs, being both biodegradable and economically viable, have emerged as the biopolymers of choice in the present context. PHAs are polymeric compounds synthesized by various bacteria, acting as carbon energy reserves.^8^ Structurally, R-hydroxyalkanoic acids serve as the monomeric form of PHAs. The categorization of PHAs is based on the number of carbon atoms in the polymeric chain, resulting in three groups: short-chain length PHAs (scl-PHAs) with 3–5 carbon atoms, medium-chain length PHAs (mcl-PHAs) with 6–14 carbon atoms, and long-chain lengths (lcl-PHAs) with more than 15 carbon atoms. The diversity of PHAs is evident in their 150 identified constituents, and combining these monomeric constituents in different proportions gives rise to polymers with varying mechanical and thermal properties.^9^ This review examines the latest information about turning the most suitable crop residues into cost-effective sustainable sources for the production of polyhydroxyalkanoate (PHA) through bacterial fermentation pathways. This review will connect missing knowledge about improved microbial strains effective pretreatment methods and workable bioprocessing approaches. The state-of-the-art incorporates research on bacterial genetics fermentation optimization and material property modifications of PHAs. Several present-day barriers exist including expensive production costs alongside unstable polymer quality indicators and insufficient financial studies for deploying PHA on an industrial scale. The solution to these gaps would transform biopolymer alternatives while enabling circular bioeconomy implementation. While over 300 bacteria have the potential to produce PHAs, only a select few have been employed for commercial production. Examples include *Ralstonia eutropha*, *Alcaligenes latus*, and *Bacillus* which can utilize various waste materials as carbon sources for PHA production. India annually generates more than 620 million tons of crop residue, yet the majority remains unutilized for livestock feed or energy production. The burning of this residue within the rice-wheat cropping system exacerbates air pollution, releasing harmful gases and impacting soil composition. Efficient management of agricultural waste not only mitigates air pollution but also provides advantageous inputs for crop development.^10^ Plants contain starch and cellulose, abundant in crop residues like corn, rice straw, and wheat straw. These materials, easily decomposed by soil microbes. Starch and cellulose, being cost-effective and readily available, are explored as alternative raw materials in the plastic ndustry for the production of biodegradable polymers.^11^ The pressing need for biodegradable polymers as substitutes for single-use plastic has led to the exploration of agricultural waste as a valuable resource. However, PHAs produced from natural carbon sources face limitations, primarily in high production costs. Raw material accounts for 40–48% of total production cost, from which 70–80% of the cost is due to carbon sources. This cost hurdle necessitates the design of new processes using waste carbon sources instead of pure ones. Some PHAs have been synthesized using waste carbon sources through an eco-friendly process, offering biodegradability and sufficient transparency. Despite heir advantages, these PHAs have drawbacks, including specific composting conditions, thermostability, brittleness, hydrophilic character, and unsatisfactory mechanical properties, especially in wet environments.^12^ For a biopolymer to be a successful substitute for synthetic plastics, it must be synthesized with a proper blend of good mechanical and thermal properties. Additionally, the synthesis and degradation processes must be economically viable. Polyhydroxybutyrate is one such biopolymer belonging to the class of PHAs, gaining attention due to its nearly identical properties to synthetic polymers. PHA boasts several advantages, including being eco-friendly, renewable, biocompatible, and biodegradable, useful for crop residue management, and biobased substitutes for synthetic polymers.^13^ The present study has been, thus designed to achieve an eco-friendly biopolymer by using microorganisms with crop residues as cheaper substrates. The abundance of crop residue worldwide presents a promising opportunity for low-cost PHA production. Nonetheless, several challenges must be tackled before commercial viability can be achieved. Utilizing crop residues for PHA production offers a dual solution to agricultural waste management and plastic pollution, contributing to a more sustainable and circular bio-economy. However, substantial research, investment, and policy support are necessary to overcome technical and economic obstacles and fully realize PHA's potential as a bio-based alternative to traditional polymers. Encouraging circular agriculture is crucial for effectively utilizing crop residues, reducing Greenhouse Gas Emissions (GHEs), and enhancing biopolymer production. Moreover, it offers economically viable opportunities to generate additional income for farmers.

This review examines the latest information about turning the most suitable crop residues into cost-effective sustainable sources for the production of polyhydroxyalkanoate (PHA) through bacterial fermentation pathways. This review will connect missing knowledge about improved microbial strains effective pretreatment methods and workable bioprocessing approaches. The state-of-the-art incorporates research on bacterial genetics fermentation optimization and material property modifications of PHAs. Several present-day barriers exist including expensive production costs alongside unstable polymer quality indicators and insufficient financial studies for deploying PHA on an industrial scale. The solution to these gaps would transform biopolymer alternatives while enabling circular bioeconomy implementation.

This review explores the potential of diverse crop residues as sustainable feedstocks for PHA production, emphasizing their role in improving yield, reducing costs, and enhancing environmental benefits. While previous studies have investigated PHA production from various waste sources, comparative analyses specifically focused on crop residues remain limited. This study provides a structured evaluation of different crop residues and their suitability for bacterial fermentation, highlighting substrate efficiency and bacteria performance in PHA biosynthesis. Additionally, this review examines PHA blending strategies to enhance mechanical properties, addressing key limitations. It also discusses economic and industrial barriers to large-scale PHA production, including cost-effective feedstock selection, process optimization, and commercial scalability. By integrating insights from biopolymer engineering, bacterial metabolism, and sustainable material development, this review provides a comprehensive framework for advancing PHA production from crop residue, supporting research to commercial application in biodegradable biopolymer.

**Research questions**.

1. Which crop residues can provide maximum PHA yield at industrial scale?

2. What reaction conditions are most suitable for optimized PHA production with least carbon footprint?

3. How can we increase the mechanical strength and thermal stability of biodegradable PHA?

4. How can we make PHA produced from agricultural waste more environmentally and economically viable as compared to petroleum-based plastics?

5. How would large scale PHA production would impact on environment as compared to petroleum-based plastics?

## 2. Environmental and health impacts of synthetic polymers

**Fig. 2:**
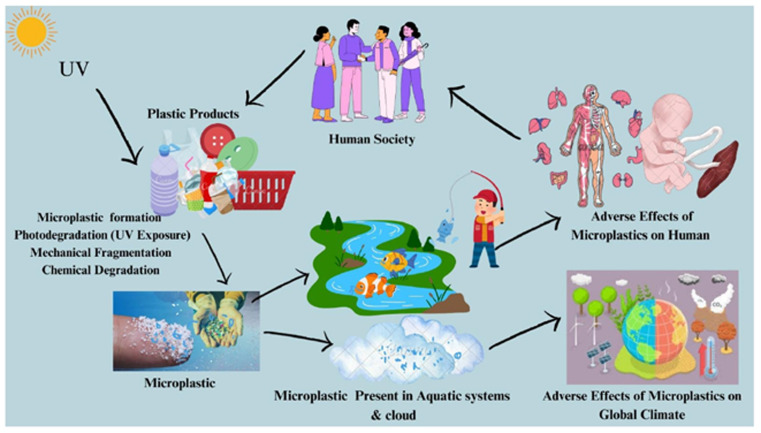
Environmental impact of synthetic polymers (The environmental and human health hazards of synthetis polymer(plastics). Plastic products undergo degradation through UV exposure, mechanical fragmentation, and chemical processes, leadimg to microplastic formation. These particles accumulate in aquatic systems, clouds, and food chains, contibuting to climate change and posing significant health risks, including developmental and organ toxicity, ultimately affecting human society).

The rise of petroleum-based plastics transformed industries due to their convenience and cost effectiveness. However, concerns about their environmental impact and sustainability are prompting a revaluation. Plastics were introduced as substitutes for traditional materials, but the cost-effectiveness of petroleum-derived feedstocks is no longer valid as non-renewable fossil fuel costs have resin. Research highlights adverse effects on human health, Aquatic systems, and air shown in Fig. 2. Phthalates are used as plasticizers in “Polyvinyl Chloride (PVC) products”.^14^ PVC, found in toys like teething and inflatable toys, poses risks due to phthalate migration into the air, food, and fetuses mentioned in Table 1. Phthalates like di 2-ethylhexyl (DEHP), Dibutyl (DBP), Diisononyl (DINP), Diisodecyl (DIDP), Benzyl butyl (BBP), and Di-*n*-octyl (DNOP) are associated with adverse outcomes like increased adiposity, insulin resistance,^15^ altered hormones, and reproductive issues in infants and children.^16^

**Table 1:** Environmental and health impacts of synthetic polymers

No.	Synthetic polymers	Environmental and health impact	Ref.
1	Polyvinyl chloride (PVC)	PVC contains toxic additives, such as phthalates, which have been linked to adverse health effects, including increased adiposity, insulin resistance, hormonal imbalances, and reproductive disorders in infants and children	15
2	Polypropylene (PP)	Polypropylene (PP) contributes to various environmental issues, including global warming, acidification, eutrophication, photochemical oxidant formation, human toxicity, and marine aquatic ecotoxicity. Additionally, the ingestion of polypropylene by animals can lead to gastrointestinal blockages, which may result in fatal outcomes	16
3	Polyethylene terephthalate (PET)	PET in air, land, and water and its associated negative health hazards on various human organs such as birth effects, cancer, liver dysfunction, and infertility	107
4	Polystyrene (PS)	Polystyrene (PS) significantly harms the environment and human health. It contributes to air and soil pollution and is associated with carcinogenesis, genotoxicity, disruptions in cell division and viability, cytotoxicity, metabolic disturbances, and DNA damage	107

Public health regulations and environmental bans now limit phthalates across countries such as Australia and Canada and all European Union members as well as the United States because of scientifically proven damage. Plastic waste pollution persists as a significant environmental challenge because of which scientists need to investigate sustainable alternatives such as PHAs even though regulatory actions exist.^17,18,107^ Plastics, including PET, contribute significantly to growing municipal solid waste and environmental challenges due to their non-biodegradability. Plastics take centuries to decompose in landfills, causing air and water pollution. Due to environmental concerns and limited land availability, landfill practices face restrictions. Thin plastic bags also clog urban drainage systems, leading to flooding. Plastic waste in oceans kills millions of marine creatures yearly, leading to bans on thin plastic bags in retail in many countries.^19^ It's vital to reconsider plastic's environmental and health impact to promote sustainable alternatives and responsible use. Polymers range from synthetic plastics (*e.g.*, polystyrene, polyethylene, polypropylene, acrylics) to natural biopolymers like Deoxyribonucleic acid, cellulose, starch, and proteins such as silk, keratin, and collagen.^20^ Synthetic polymers are formed through chemical polymerization, where monomers combine into larger units, resulting in organic polymers with greater molecular mass, known as plastics.^21^ Synthetic polymers have longer chained than natural ones, imparting unique properties like strength, flexibility, and light weightiness'. Starch, a natural polymer composed of glucose, is abundant in plants and serves as their carbohydrate reserve. It belongs to the family of natural biopolymers called “polysaccharides, which are Earth's most prevalent biopolymers. Examples of polysaccharides include “cellulose, glycogen, and chitin. Starch is versatile and finds applications in polymer technology, as it can be converted into chemicals like ethanol, acetone, and organic acids. It also contributes to the production of synthetic polymers and can be used to create biopolymers through fermentation or hydrolysis for monomer and oligomer production.^22^ Additionally, it can be grafted with various reagents to create new polymeric materials, either as standalone substances or additives in other polymers.^23^ Plastic production has seen a remarkable surge in the past decade, surpassing the entire production of the previous century. Global plastic production increased from 322 million tonnes in 2015 to 335 million tonnes in 2016.^24,25^ With the growing demand for plastic materials, there is a rising need to explore environmentally friendly alternatives to traditional petroleum-based polymers. This drive for more eco-friendly options has spurred scientists to develop biobased or biodegradable polymers capable of controlled degradation in specific environments.^26,27^ The pollution problems stemming from environmental contamination increase because synthetic polymers made from petrochemicals exist as non-biodegradable substances. Biological systems contain synthetic polymer remnants that build microplastic levels and damage both sea and land organisms. An ecological transition needs to occur through the implementation of biopolymers because they diminish environmental impact compared to current alternatives.

### 2.1 Country-wise regulations

The commercial expansion and uses of polyhydroxyalkanoates (PHAs) face different regulatory rules depending on the country which leads to concerning consequences for their use. According to European Green Deal regulations and REACH (Registration, Evaluation, Authorisation, and Restriction of Chemicals) policies the EU supports biopolymer development of PHAs. The production expenses remain high while the scalability remains restricted. The Food and Drug Administration grants permission for PHAs in food packaging applications and healthcare but PHA adoption remains restricted by its higher cost compared to traditional petroleum plastics. Japan uses government-driven investments together with strategic alliances to develop PHA technology specifically in biomedical applications but this advance is restricted by premium feedstock costs. Emerging economies like India and China face regulatory ambiguity, with insufficient infrastructure for PHA waste management and high manufacturing expenses.^116^ Standardized regulations should be the global priority while efforts must be made to decrease production costs using waste materials alongside the creation of strong biodegradability criteria for making PHA commercially viable in numerous markets.

## 3. Physicochemical properties of biopolymers

Biopolymer properties can be classified into three categories: relative properties, synthesizing properties, and component properties. Relative properties encompass inherent attributes like density, solubility, transparency, and permeability. Synthesizing properties pertain to quality parameters such as viscosity, optical purity, mechanical properties, stability, and molecular weight. Component properties combine these attributes, directly impacting the performance and functionality of biopolymers.^28^ Stereo complexes of biopolymers play a pivotal role in enhancing the utility of fibers and enabling controlled drug release, tissue engineering, and various medical applications. The characterization of both non-degradable and biodegradable biopolymers has unveiled intricate interactions influenced by stereoselective van der Waals forces, which affect the physical properties of the synthesized biopolymers.^29^

Physical properties of biopolymers and their composites include characteristics like melting and boiling points, shape, density, and viscosity. Interaction with water can alter their internal structure, making them moisture-sensitive. Hydrophilic biopolymers, like cellulose and hemicellulose, swell as water molecules adsorb, reducing hydrogen bonds and weakening their physical and mechanical properties. Thermal properties depend on factors like “thermal stability and conductivity, with parameters such as degradation rate, glass transition temperature, melting point, crystallization temperature, crystallinity, and heat of fusion Some Properties of different polymers are analyzed using techniques like Differential Scanning Calorimetry and Thermogravimetric Analysis.^30^ Mechanical properties of biopolymer composites depend on factors like temperature, applied force, deformation, and heating/cooling rates, customized for specific applications. These conditions influence the mechanical characteristics of biopolymers and their composites. Various mechanical properties are vital for biopolymer material design, including Young's modulus, tensile strength, Poisson's ratio, rheology, and elongation at break, with relevance in industrial sectors.^31^ Tensile and impact strength are primary techniques for characterizing fiber-reinforced composites. Optical properties are crucial in various industrial applications like coatings, plastics, and transparent materials, especially in preserving food quality by protecting against UV exposure and lipid oxidation^32^ Key optical parameters studied include colour, transparency, polarizability, light absorption/transmission, absorption coefficient, dielectric properties, and refractive index. Polymers vary in colour, transparency, and opacity, each suited for specific applications in the food and pharmaceutical industries. The refractive index influences light refraction, and polarizability relates to molecular weight and biopolymer identity.^33^ Biopolymers demonstrate multiple physicochemical features including full decomposability together with high temperature tolerance as well as bio friendliness to human tissues. The properties of melting point and crystallinity and tensile strength become adjustable through structural changes. The suitable characteristics of these materials allow their use in biomedical devices along with packaging solutions and agricultural applications where they function as environmentally friendly alternatives to plastics.

## 4. Manufacturing techniques of polyhydroxyalkanoates (PHAs) from crop residue

The manufacturing of PHAs involves several key steps, including substrate (crop residue) selection, bacterial strain selection, carbon source pretreatment, fermentation, biomass separation from the broth, biomass drying, PHA extraction, PHA drying, and packaging. The sustainability of PHA production, its ability to utilize diverse crop residue as substrate, and its operation under moderate process conditions underscore its potential as an environmentally friendly alternative to conventional plastics.^117^

### 4.1 Substrate selection and pretreatment

The initial stage of PHA production from crop residues involves pretreatment to enhance carbohydrate accessibility. In the case of crop residue, complex carbohydrates are converted into sugar monomers using enzymatic hydrolysis, which is subsequently fermented by bacteria to produce PHAs. However, due to the structural complexity of crop residue substrate, composed of cellulose, hemicellulose, and lignin, it exhibits high recalcitrance. To overcome this challenge, pretreatment techniques such as mechanical, chemical, and oxidative methods are employed. These methods break down the crop residue structure, making cellulose more accessible to enzymatic hydrolysis and increasing sugar yield for microbial fermentation.^118^

### 4.2 Bacteria selection

Bacteria Strains for PHA Production A diverse range of bacterial species, exceeding 300, have been reported to produce PHAs under various environmental conditions, including both aerobic and anaerobic environments. PHAs have been synthesized by both Gram-positive and Gram-negative bacteria, with the most extensively studied strains being *Bacillus megaterium*, *Ralstonia eutropha* (currently *Cupriavidus necator*), and *Pseudomonas putida*. Other notable genera include *Azotobacter*, *Syntrophomonas*, *Aeromonas*, and *Clostridium*.^119^ The selection of an appropriate bacteria for industrial-scale PHA production is determined by factors such as growth rate, polymer synthesis efficiency, polymer quality and quantity, and the maximum achievable polymer accumulation. Fermentation and PHA Biosynthesis during fermentation, selected bacterial strains utilize the pretreated crop residue as a carbon substrate, synthesizing PHA molecules intracellularly. The efficiency of fermentation depends on process parameters such as nutrient concentration, aeration, temperature, and pH control. Optimizing these parameters ensures maximum polymer yield while maintaining cost-effectiveness for large-scale production. After fermentation, PHAs undergo several essential downstream processing steps to ensure high recovery efficiency and purity.

### 4.3 Cell lysis and centrifugation

The bacterial cells accumulate PHAs intracellularly in granules as carbon and energy reserves. To extract the polymer, biomass is separated from the fermentation broth using centrifugation, followed by cell lysis to release the trapped product.

### 4.4 Solvent extraction

Various solvents, including cold methanol and chloroform, have been utilized for PHA recovery. The polymer remains in the cell debris post-lysis and can be efficiently extracted using these solvents. Recent advancements have explored the use of non-halogenated solvents such as ethyl acetate and methyl isobutyl ketone, which have demonstrated up to 99% purity while reducing production costs and environmental impact.

### 4.5 Membrane filtration and purification

Following solvent extraction, membrane filtration is employed to segregate PHAs from other small biomolecules present in the extract. This step ensures the removal of unwanted contaminants and enhances polymer purity.

### 4.6 Quantification and characterization

The recovered PHA product is quantified and analyzed using techniques such as Gas Chromatography (GC) to determine yield and purity. Additional characterization methods include Nuclear Magnetic Resonance (NMR) spectroscopy and Flow Cytometry, which confirm the molecular structure and composition of the polymer.^120^

PHA production from crop residue substrates offers a sustainable and biodegradable alternative to synthetic plastics. The integration of optimized pretreatment techniques, efficient microbial fermentation, and advanced downstream processing methods has significantly improved PHA yield and purity. Continued research into alternative feedstocks and eco-friendly extraction techniques will further enhance the commercial viability of PHAs as a green biopolymer.

### 4.7 Crop residue as a carbon source for PHA production

**Fig. 3:**
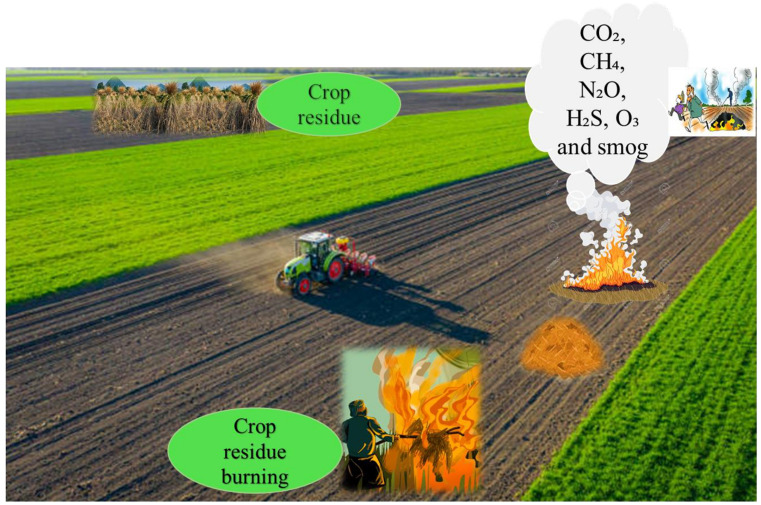
Burning of crop residue causes air pollution (The burning of crops residue releases harmful gases, including CO_2_, CH_4_, N_2_O, H_2_S, O_3_ and smog, which significantly contribute to air pollution and pose serious risks to public health).

Second-generation biomass consists of various non-edible bio-wastes that provide an ethically acceptable and widely accessible feedstock. Over 1 billion tonnes of agricultural and food waste are generated each year. The practice of burning fields to clear crop residue for the timely planting of the next crop is common, due to the low nutritional value of the residue and the high labor costs associated with field clearance. However, these burning releases harmful gases such as CO_2_, CH_4_, N_2_O, H_2_S, O_3_, and smog, shown in Fig. 3 which have a significant negative impact on public health and disrupt soil properties by destroying beneficial microorganisms.

Efficient management of agricultural waste helps reduce air pollution and enhance crop yields.^10^ Plants contain starch and cellulose, both of which can be used for the production of biodegradable polymers.^11^ Cellulose, the most abundant biomolecule on Earth, is widely available at a low cost.

Crop residues such as corn, rice straw, and wheat straw are rich in cellulose. Both starch and cellulose can decompose through the action of soil microbes without harming the ecosystem, making them ideal for biodegradable polymer production.^34^

The carbon source is pivotal in polyhydroxyalkanoates (PHA) synthesis. Utilizing inexpensive raw materials like residues from the agro-industry for PHA biosynthesis is an effective strategy. Various bacterial strains and substrates such as wheat straw, rice straw, and sugarcane bagasse are used in PHA production. Crop residues, rich in cellulose and hemicellulose, can be hydrolyzed into fermentable sugars for PHA conversion, as outlined in Table 2. Given that crop residues serve as a feedstock for PHA production, understanding their quantity is crucial. The production of biodegradable polymer using agricultural waste is vital for both environmental and human health. Agricultural waste products can significantly contribute to the production of biopolymers, thus promoting environmental sustainability. This section underscores the diverse crop residues that can serve as potential renewable feedstocks for PHA production.

**Table 2:** Chemical composition of various crop residues^a^

Crop residue	Cellulose (%)	Hemicellulose (%)	Lignin (%)	Ref.
Corn stover	37.6	21.5	19.1	96
Rice husk	34.7	17.4	25.5	94
Cornstalk	34.5	27.6	21.8	95
Wheat straw	32.7	24.5	16.8	97
Wheat husk	36	18	16	98
Sorghum straw	35.4	19.4	10.3	99
Sugarcane bagasse	26–47	19–33	14–23	100
Soybean hulls	29–51	10–25	1–4	101
Sunflower stalk	35–45	5–10	3–5	102
Teff straw	41.8	38	17	103
Cottonseed husk	30–32	—	17–19	106
Mustard stalk	48.5	29.6	—	104
Millet straw	36.68	17.31	19.38	105

a (The percentage of cellulose, hemicellulose, and lignin in various crop residues).

### 4.8 Polyhydroxyalkanoate (PHA) production from rice residues

India stands as the world's second-largest rice producer, with an annual output of 125.039 million tonnes. Rice residue, comprising 34.7% cellulose, 17.4% hemicellulose, and 25.5% lignin, represents a valuable raw material for PHA production. Numerous studies, as outlined in Table 3.,^35^ have investigated the potential of rice residues as a carbon source for PHA synthesis.^36^ Among these, *Bacillus firmus* NII 0830 demonstrated the highest PHA yield at 89%.^46,47^ Additionally, other *Bacillus* species, such as *Bacillus megaterium* B-10, have shown the ability to convert rice straw into high-value polyhydroxyalkanoate (PHA), underscoring the versatility of these microorganisms in biopolymer production. PHA production reached 32.56%, with a cell dry weight (CDW) of 4.67 g L^−1^.^41^ Narayanan *et al.* investigated the use of *Bacillus mycoides* DFC1 to synthesize P3HB-*co*-3HV from rice husk, yielding a polymer concentration of 0.9 g L^−1^ and a polymer content of 21.6%.^40^ In comparison, two other *Bacillus* species, *Bacillus cereus* strains VK92 and VK98, demonstrated significantly better performance with rice straw as the substrate, producing PHA contents of 59% and 46.4%, respectively.^45^ Recent studies show that various microorganisms in activated sludge can produce polyhydroxyalkanoate (PHA) using rice straw hydrolysate as a substrate.^37–39^ In contrast, Saratale and Oh demonstrated that *Ralstonia eutropha* ATCC 17697, when fed with pretreated 2% NaOH rice straw, achieved the highest PHB concentrations of 9.8 g L^−1^ and a polymer content of 70.1%.^45^

**Table 3:** Polyhydroxyalkanoate (PHA) production from rice residue and bacteria

Crop residue	Bacteria	PHA	CDW (g L^−1^)	PHA (g L^−1^)	PHA yield (%)	Ref.
Rice husk	*Bacillus mycoides DFC1*	P (3HB-*co*-3HV)	—	0.39	21.6	40
Rice husk	*BurkhaderiacepacianUSM JCM 15050*	PHB	—	4.85	40.0	40
Rice straw	*Bacillus cereus VK92*	—	5.00	0.062	59.3	45
Rice straw	*Bacillus cereus VK98*	—	5.42	0.052	46.4	45
Rice straw	*Bacillus firmus NII 0830*	—	1.90	0.019	89.0	46 and 47
Rice straw	*Cupriavidus necator ATCC 17697*	—	1.71	0.03	21.0	45
Rice straw	*Cupriavidus necator ATCC 17697*	—	1.59	0035	53.0	45
Rice straw	*Ralstonia eutropha MTCC 1472*	—	19.2	—	37.55	48 and 49
Rice straw	*Ralstonia eutropha ATCC 17697*	PHB	15.5	0.206	70.1	45
Rice straw	*Bacillus megaterium B-10*	PHB	4.67	1.496	32.56	41
Rice husk	*NK13*	—	1.01	—	11.20	48 and 49
Rice husk	*Priestia JY310*	PHB	6.2	3.1	50.4	42

Polyhydroxybutyrate or PHB is a natural polymer which is synthesised by bacterial fermentation process. It is biodegradable thermoplastic thus making it a better choice than conventional plastics materials. PHB is an incredibly strong, thermally stable, and biocompatible material applicable in packing, agriculture and medicine making thus creating a positive impact on sustainability of materials used. Ahn *et al.* used the same strains R. ATCC 17697 for PHA production from pretreated 6% H2SO4 and 2% H2SO4 rice straw as a substrate for PHA production. Achieved PHB contents of 21% and 53.0%.^45^ Another *R. eutropha* MTCC 1472 yielded a PHA accumulation of 37.55% and cellular dry weight (CDW) of 19.2 g L^−1^ from the rice straw.^48–51^ The researchers utilized rice husk hydrolysate as a carbon source to assess the PHA production potential of 10 isolated microbial strains. Their results revealed that the NK 13 strain produced the lowest biomass at 1.01 g L^−1^, with a PHA content of 11.2%.^48–50^ Priestia sp. strain JY310 demonstrated PHA production of 50.4% and a cell dry weight of 6.2 g L^−1^, further highlighting the potential of using inexpensive rice husk as a sustainable and economically viable feedstock.^42–44^ Another Priestia species, Priestia megaterium POD1, also utilized rice husk for PHA synthesis.^53^ Additionally, Vigneswari *et al.* reported that Burkholderia cepacia JCM 15050, when fed with rice husk hydrolysate, achieved a PHB content of 40%.^40^ While rice residue offers a promising avenue for PHA production, several challenges hinder its widespread use. The complex structure of lignocellulosic biomass necessitates pre-treatment before rice residue can be utilized as a carbon source for PHA manufacturing. Further research is needed to identify bacteria capable of utilizing the entire lignocellulosic biomass for maximum PHA production, ideally without the need for pre-treatment.

### 4.9 Polyhydroxyalkanoate (PHA) production from wheat residues

Wheat straw is another promising, cost-effective carbon source for PHA production, containing glucose, xylose, and arabinose as its main sugars. Hydrolysate wheat straw as a source of carbon with *Burkholderia sacchari* DSM 17165 achieved 0.7 g L^−1^ P (3HB-*co*-4HB).^52^ Two *Cupriavidus necator* strains produced the most promising results when cultivated on wheat straw. *Cupriavidus necator* ATCC 17699 and *Cupriavidus necator* DSM 545 achieved PHB contents of 65% PHB, 15.3 g L^−1^ biomass and 80.1% PHB, 12.1 g L^−1^,^54,55^ Strains of *Cupriavidus necator* can produce polyhydroxyalkanoate (PHA) from wheat straw, most notably polyhydroxybutyrate. According to Zhang, *et al.*, Glucose and xylose from wheat straw can be combined to substitute additional carbon sources for the production of PHB. Some PHA syntheses from wheat residues are mentioned in Table 4. These findings suggested research on an integrated biorefinery incorporating the production of PHA on a commercial scale using wheat straw and the conversion of wheat straw into two major products, hydrogen and polyhydroxyalkanoate using *C. necator* DSM 545. Higher economic investment, technological improvement, and financial subsidies from government agencies are required for the large-scale operations of such integrated systems. Further comparative research on the PHA generation from wheat straw is still required.

**Table 4:** Polyhydroxyalkanoate (PHA) production from wheat residues and bacteria

Wheat residue	Bacteria	PHA	CDW (g L^−1^)	PHA (g L^−1^)	PHA yield (%)	Ref.
Wheat straw	*Burkholderia sacchari DSM 17165*	P (3HB-*co*-4HB)	—	0.7	—	52
Wheat straw	*Cupriavidus necator ATCC 17699*	PHB	15.3	—	65	54
Wheat straw	*Cupriavidus necator DSM 545*	PHB	15.1	0.252	80.1	55

### 4.10 Polyhydroxyalkanoate (PHA) production from corn residues

In 2023, approximately 1.22 billion tonnes of corn were produced, representing 31% of the world's total cereal production. In conventional agriculture, corn residues including stalks, leaves, husks, and cobs left in the field after harvest—are often regarded as waste resources. These waste materials are used to produce biopolymers through microorganisms, as outlined in Table 5. Paracoccus sp. LL1 utilizes corn stover, resulting in a polyhydroxyalkanoate (PHA) content of 72.4% and a production level of 9.71 g L^−1^. The yield of PHA from corn stover surpasses that obtained from pure glucose, xylose, and cellobiose.^56^ In addition, Hierro *et al.* investigated *Bacillus* sp. produced PHB from corn cobs, demonstrating a yield of 51% PHB accumulation with a concentration of 4.80 g L^−1^.^57^ Another *Bacillus* sp. *B. megaterium* Ti3 demonstrated the ability to produce 57.8% PHB concentration within 48 hours using pretreated corn husk hydrolysate, interestingly *Bacillus megaterium* Ti3 showed even better results.^61–64^ Study by Salvachúa *et al.*, *P. Putida* KT2440 was utilized for PHA production using corn stover, resulting in PHB accumulation of 17.6% with a concentration of 1.00 g L^−1^.^58,59^ Khomlaem, *et al.*, reported that the various approaches to the production of polyhydroxyalkanoates (PHAs) like PHB and its copolymers using different microbial strains and the carbon resources of corn cob and corn straw are pointed out in the research stated above. B. megaterium ALA2 and Paracoccus sp. LL1:- These are the cell retention culture used in corn cob carbon source to produce high concentration PHA, at 57.6 ± 0.18 g L^−1^, 56.62% of dry cell weight and 31.3 ± 0.23 g L^−1^ 44.5% of the dry cell weight. *Cupriavidus necator* DSM 545: This strain was used to produce P (3HB) from corn stover alkaline pretreatment fluid containing oxidative enzymes, mediators, surfactants and silicon nanoparticles and the obtained PHB concentration was 3.3 g L^−1^. Azohydromonas lata DSM 1122 and B. sacchari DSM 17165: These two were tested for production of PHA biopolymers using blends of corn stover hydrolysate and levulinic acid. Dairy waste microbial culture: This culture was able to ferment corn straw into PHBV and the yield was 41.4% within 72 h in shake flask and 76.3% within 96 h in stirred tank bioreactor. Arranged *E. coli* WJ03-02: This strain was employed for the production of P3HB-*co*-LA from corn stover hydrolysate. Reh06 Strain: This strain has the ability of fermenting glucose, xylose, *p*-coumaric acid and ferulic acid in corn stover hydrolysates yielding 11.51 g L^−1^ of polyhydroxybutyrate. These works show that lignocellulosic biomass can be used as carbon substrate for PHA synthesis through the use of both wild and genetically modified microorganisms. This makes it evident that there is continuous research on the various procedures of enhancing the production of PHA for use in industries, ranging from microbial cultures to genetic engineering.^65^

**Table 5:** Polyhydroxyalkanoate (PHA) production from corn residues and bacteria

Corn residue	Bacteria	PHA	CDW (g L^−1^)	PHA (g L^−1^)	PHA yield (%)	Ref.
Corn stove	*Pseudomonas putida KT2440*	PHA	5.68	1.00	17.6	58
Corn stover	*Paracoccus* sp*. LL1*	PHB	—	9.71	72	56
Corn cob	*Bacillus* sp	PHB	—	4.80	51.6	57
Corn husk	*Bacillus megaterium Ti3*	—	1.7	1.0	57.0	61
Corn stover	Cupriavidus *necator DSM 545*	PHB	—	3.3	—	60

Polyhydroxyalkanoate (PHA) can be efficiently produced from corn residues such as corn cobs, husks, and stover. Remarkably, Paracoccus sp. LL1 was the highest PHA production 72% from corn stover, indicating its potential as a promising PHA producer from crop residues.

### 4.11 Polyhydroxyalkanoate (PHA) production from Sugarcane Residues

Sugarcane bagasse, containing 26–47% cellulose, 19–33% hemicellulose, and 14–23% lignin, is a valuable resource. Each year, approximately 540 million tonnes of sugarcane bagasse (SB) are produced across 124 countries. The production of PHA using Sugarcane Residues as a substrate and various strains of bacteria are mentioned in Table 6.^66^ de Souza and colleagues demonstrated that *Bacillus megaterium* Ti3 using sugarcane bagasse, accumulated 0.88 g L^−1^ of dry cell weight and 65% PHA.^67^ study by Paula *et al.*, B. glumae MA13 showed PHA synthesis using inexpensive feedstocks.^68^ Additionally, Paula *et al.* achieved a PHA content of 65% w/w in *C. necator* with a C/N ratio of 20 when cultured on dilute acid-pretreated sugarcane bagasse.^68^ Saratale, *et al.*, used sugarcane bagasse hydrolysate, B. cepacia IPT 048 yields 53% of cell dry weight (CDW) and a concentration of PHB at 2.3 g L^−1^. On the other hand, B. sacchari IPT 101 shows a greater percentage of PHB production, producing 62% PHB.^69^ Burkholderia F24 bacteria used sugarcane bagasse to produce 3- hydroxybutyrate.^70^

**Table 6:** Polyhydroxyalkanoate (PHA) production from sugarcane residues and bacteria

Sugarcane residue	Bacteria	PHA	CDW (g L^−1^)	PHA (g L^−1^)	PHA yield (%)	Ref.
Sugarcane bagasse	*Burkholderia glumae MA13*	—	—	0.09	14.95	68
Sugarcane bagasse	*Burkholderia* sp*. F24*	PHB	25.04	0.28	49.31	70
Sugarcane bagasse	*Burkholderia cepacia IPT 048*	PHB	—	2.3	53	69
Sugarcane bagasse	*Burkholderia sacchari IPT 101*	PHB	—	2.7	62	69
Sugarcane bagasse	*Bacillus thuringiensis*	PHB	—	4.2	39.6	73
Sugarcane bagasse	*Ralstonia eutropha* + *Lysinibacillus* sp/*Co-culture*	—	—	6.38	70.0	69
Sugarcane bagasse	*Bacillus megaterium Ti3*	—	0.88	0.58	65	67
Sugarcane bagasse	*Lysinibacilllus* sp	—	8.65	5.31	61.5	72
Sugarcane bagasse	*Ralstonia eutropha*	—	—	6.06	56.7	75

Saratale, *et al.* described the possibility of efficiently producing high-value polyhydroxyalkanoates (PHA) from sugarcane bagasse by using a microbial co-culture approach^69^ Attapong, *et al.*, successfully produced PHB through the bioconversion of sugarcane bagasse hydrolysate.^71^

Lysinibacilllus sp. has been used to obtain 8.65 CDW with 61% of PHB from Sugarcane Bagasse hydrolysates.^72^ The PHA produced using Sugarcane Bagasse substrates as feedstocks was yield of 5 39.6% PHB accumulation with a concentration of 4.2 g L^−1^.^73^

Numerous studies have explored the biosynthesis of polyhydroxyalkanoates (PHA) using sugarcane bagasse as a substrate. Remarkably co-culturing *Ralstonia eutropha* and *Lysinibacillus* sp. yielded a PHA accumulation rate of 70% co-culture as promising approaches for enhancing PHA productivity. Further research on microbial consortia is important to optimize PHA production for large-scale applications. Advancing research on microbial consortia is key to improving large-scale PHA production processes.^74^

### 4.12 Polyhydroxyalkanoate (PHA) production from other crop residues

According to Silambarasan *et al.*, P (3HB) production from finger millet straw using *Bacillus megaterium* strain CAM12 showed the best results and was able to accumulate 8.3 g L^−1^ of PHB at a 51.8% polymer content.^76^ On the other hand, According to Naitam *et al.*, when *Bacillus* sp. was explored using teff straw, the PHA production was 37.4% of cell dry weight (CDW) with 8.3 g L^−1^.^70^ The first successful production of polyhydroxyalkanoate (PHA) by utilizing soybean hull hydrolysate as a carbon source *C. necator* strain DSMZ545 was used in this process yielding PHB 31.8%.^77^ The recombinant *R. eutropha* strain with the *E. coli* XylAB genes was tested on sunflower stalk hydrolysate and produced 72% PHB.^78^*P. aeruginosa* was screened for PHA production from Cottonseed husk producing PHB contents of 0.26%.^70^ This section summarises five different crop residues as a substrate used for PHA production mentioned in Table 7. The highest yield of 72% of dry cell weight was obtained with sunflower stalk Other substrates with decreasing yield are finger millet straw 51% Teff Straw 37.4% soybean hull 31%, and Cottonseed husk 0.26% PHB production. Although crop residue is an attractive source of PHA production there are several challenges to its widespread use. Because crop residue has different chemical compositions, pre-treatment is required before fermentation. Inhibition during fermentation, achieving high PHA yields, and improving PHA recovery processes. The economic viability of crop residue-based PHA synthesis at Mass production in these areas needs to be further developed. Crop residues serve as an economical and sustainable option to produce the polymer polyhydroxyalkanoate (PHA). The studies prove that fermentable sugar-rich rice straw hydrolysates enable efficient growth of *Bacillus* and *Cupriavidus necator* bacteria for PHA synthesis production. The modification of pretreatment processes enhances both sugar production rates and removes harmful substances which leads to better polymer accumulation. Acid or enzymatic treatment of wheat straw generates both glucose and xylose. The fermentation process of *Ralstonia eutropha* utilizes these sugars to produce PHA while co-culture solutions boost the quantity and quality of the obtained polymers. The hydrolysates made from corn stover serve as excellent raw materials for *Cupriavidus necator* to produce high amounts of PHA. Fed-batch fermentation together with production efficiency increases leads to a process that reduces operational costs while minimizing ecological effects. Sugarcane bagasse provides an excellent source for fermentable sugar extraction since pretreatment procedures break down cellulose and hemicellulose present in this agricultural waste substance. Microorganisms from the species *Halogeometricum and Lysinibacillus* use fermentation to produce significant PHA concentrations which may become the foundation of bioplastics and packaging materials. The investigation into sustainable PHA synthesis includes additional crop residues which include soybean hulls alongside barley straw and cassava peels. Such biological feedstocks contain necessary nutrients while decreasing manufacturing expenses which makes them useful for sustainable PHA production.

**Table 7:** Polyhydroxyalkanoate (PHA) production from other crop residues and bacteria

Crop residue	Bacteria	PHA	CDW (g L−1)	PHA (g L−1)	PHA yield (%)	Ref.
Finger millet straw	*Bacillus megaterium strain CAM12*	PHB	16.23	8.31	51	76
Soybean hull	*Cuparividus necator strain DSMZ 545*	PHB	—	—	31	77
Sunflower stalk	*Ralstonia eutropha*	PHB	—	7.86	72	78
Cottonseed husk	*Pseudomonas aeruginosa*	PHB	—	—	0.26	70
Teff straw	*Bacillus* sp	PHB	8.3	3.2	37.4	70

## 5. PHA modification *via* blending

Blending is an effective method for creating improved polymeric materials that can address the limitations of the parent components. By carefully selecting starting materials, adjusting compositions, and optimizing preparation conditions, the physical and mechanical properties of polymer blends can be tailored”. The inclusion of biodegradable components enhances attributes like complete biodegradability and biocompatibility. “These blended materials offer numerous applications, from environmentally degradable resins to biomedical implants and surgical sutures. Incorporating PHAs into blends with other biodegradable polymers has the potential to enhance PHAs' overall performance”.^79^ Some blending agents and PHA are mentioned in Table 8. Starch, a biodegradable and renewable natural polymer, offers significant promise due to its abundance. Comprising amylose and amylopectin molecules, it has been extensively studied in combination with PHA, particularly PHB, to enhance properties and reduce costs. McAdam *et al.*^80^ investigated the compatibility of PHB and starch blends, discovering that these blends exhibited a single glass transition temperature (Tg) and were crystalline. Notably, a blend with a 30 : 70% ratio showed a significant increase in tensile strength compared to pure PHB. By incorporating starch into PHB at a maximum of 30%, it's possible to substantially reduce PHB's overall cost while preserving its physical attributes. These blends could find applications as coating materials for food packaging on paper or cardboard substrates.^81^

**Table 8:** Blending agents and PHA

PHA type	Blending agent	Modification effects	References
PHB	Starch	Improved flexibility and elongation, reduced brittleness	85
PHB	Poly (ethylene oxide)	Enhanced mechanical properties, increased water absorption	85
PHB	Polyvinyl alcohol	Improved film transparency, increased flexibility	85
PHB	Chitosan	Increased water vapor barrier properties, reduced water absorption	86
PHBV	Polycaprolactone (PCL)	Enhanced flexibility, improved tensile strength	87
PHBV	Poly (ethylene glycol)	Increased hydrophilicity, improved film transparency	88
PHBV	Polyvinyl alcohol (PVOH)	Enhanced film flexibility, reduced water vapor permeability	85
PHBV	Starch	Improved water absorption and mechanical properties	85

Cellulose derivatives, which include “ethyl cellulose (EC), cellulose propionate, and cellulose acetate butyrate (CAB), are versatile biomaterials widely utilized in various applications, such as blood coagulants, pharmaceutical tablet coatings, and carriers for poorly soluble drugs.^82^ Notably, cellulose derivatives are susceptible to enzymatic digestion within the human body. These derivatives have attracted considerable interest as potential blending components with PHA (polyhydroxyalkanoates) due to their compatibility with PHA and their ability to enhance PHA degradation. A study conducted by Zhang *et al.*^83^ investigated the miscibility, crystallization, melting behaviour, and phase morphology of PHB (polyhydroxybutyrate)/EC blends. Their findings revealed that the glass transition temperature (Tg) was dependent on the blend composition, with an increase in Tg observed as the PHB concentration in the blends decreased. Unlike pure PHB, the blends did not crystallize when cooled from a molten state during non-isothermal crystallization runs in differential scanning calorimetry (DSC). Furthermore, the presence of EC significantly delayed the growth of PHB spherulites within the blends. Furthermore, it's worth noting that blends involving different types of PHA members are typically compatible and offer an effective means of enhancing co-crystallization. For instance, Yoshie *et al.* conducted a study on the solid-state structures and crystallization kinetics of PHBV and PHB/PHBV blends”.^84^ Their findings revealed several observable phase structures, including (1) Co-crystallization of PHB and PHBV. (2) Coexistence of two or more crystalline phases resulting from phase segregation during crystallization. (3) Immiscible phase separation, which undergoes changes with an increase in the HV content of PHBV. As the HV content increased, phase segregation became more pronounced before co-crystallization occurred. Consequently, the copolymer content within the co-crystalline phase decreased, and crystalline phases of the individual polymers were formed. Polyhydroxyalkanoate poor mechanical and thermal properties restrict them from becoming widely used in high-value applications. Enhancing PHAs' mechanical and physical properties while providing their thermostability and biodegradability without posing a threat to the environment must be the main goal of current research. Blending is a process which is used in reinforced the polymeric materials using its parent components to achieve different physical and mechanical characteristics. These polymer blends can be altered by using appropriate materials, varying the composition of the blend and optimizing the conditions that can be used in the process. It may be indicated that the utilization of biodegradable constituents like PHAs (polyhydroxyalkanoates), and the natural polymers: starch or cellulose will help in the improvement of biodegradability, biocompatibility while decreasing cost. For example, using blends of PHB with other materials exhibits better tensile strength and low cost while ethyl cellulose which is a cellulose derivative can also be incorporated into PHA to help in degradation and alteration of physical characteristics. Blends that include such structures as potential applications in degradable resins, biomedical implants, as well as in packaging materials. These composites have displayed their properties such as coalescing single Tg as well as a different kind of crystallization and this has created room in material science. Researchers enhance PHA properties by combining these materials with starch or cellulose derivatives along with polylactic acid to achieve improved strength as well as excellent durability and affordability. Through modifications PHA performs better in flexible applications as well as degrades easily which expands its potential use in medical devices and food packaging and agricultural films. Blended materials solve two PHA drawbacks with brittleness and low thermal resistance.

The blending process enhances polyhydroxyalkanoates (PHA) properties because it solves both mechanical strength, weaknesses and stability problems. Manufacturers should use compatible biodegradable substances while tuning their blending parameters for obtaining preferred material properties. PHA blending occurs most often with starch and cellulose derivatives because these materials possess abundant availability and low cost combined with their ability to increase product biodegradability and mechanical strength. Starch-PHA blends are particularly notable. The cost-effective components of starch contain amylose together with amylopectin to enhance tensile strength. The combination of PHB and starch in a 30 : 70 blend presented enhanced tensile strength.^108^ These blends find their most suitable applications in food packaging through their use as paper and cardboard coatings that present sustainable and affordable alternatives. The incorporation of ethyl cellulose (EC), cellulose derivatives helps enhance degradation together with thermal property modification. PHB/EC blends showed delayed spherulite evolution together with one-of-a-kind crystallization patterns which implied better thermal properties.^109^ Different PHA types in combination demonstrated constructive results for potential applications. PHBV and PHB/PHBV blends revealed co-crystallization and phase segregation that impacts the mechanical and thermal properties of the material.^110^ The modified products now reach wider application areas in medical fields including both implant technology and surgical thread usages.

Marketed Products & Cost-Effectiveness: The blend of PHA functions as an environmentally friendly material for biomedical implements in addition to biodegradable food packaging. The market affordability results from combining costly PHAs with lower-priced natural polymers while maintaining vital material characteristics.^111–113^

Advantages & Disadvantages: Using PHA blends results in three beneficial outcomes such as stronger materials and easier decomposition and cheaper manufacturing. The existence of phase separation issues and restricted thermal reliability continues to require additional study because of these disadvantages.^114^

Other Modification Techniques: The modification methods observed beyond blending include grafting, copolymerization and plasticization. Grafting enables the addition of flexible side chains yet copolymerization enables better performance through changes in crystalline structures. The introduction of plasticizers enhances material workability although it lowers thermal resistance levels. Spirit blending stands as a budget-friendly process for PHA modification and more research is underway to boost performance in different application sectors.^115^

## 6. Barriers to implementing PHA biopolymers

The PHA production price is still far above the price of conventional plastics. To make the process economically attractive, many goals have to be addressed simultaneously.

Commercially available biopolymers are expensive due to their raw materials and processing/manufacturing high cost. The primary obstacle to the widespread adoption of PHAs across industries is their elevated cost. While synthetic plastic production averages around $1250 USD/Mt, PHA production ranges from $4000 to $15000 USD/Mt. This substantial disparity in costs poses a significant barrier for businesses requiring large volumes of polymers annually. Substrates are major costs in PHA production. If low-cost substrates, such as Crop residue, food waste, treated kitchen waste, and heat treated activated sludge, algae could be used, PHA production costs would be significantly reduced. Recombinant microbial strains are being developed to achieve both a high substrate conversion rate and close packing of PHA granules in the host cell.^89^ A more efficient fermentation process,^40^ better recovery/purification,^90^ and the use of inexpensive substrates^91^ can also substantially reduce the production cost”. Recently, extremophilic bacteria, particularly the halophilic Halomonas spp., have emerged as promising candidates for reducing costs and scaling up the production of PHAs. Novel genetic engineering strategies based on extremophiles have been devised to address the significant challenges associated with commercializing PHAs, particularly those linked to cost–intensive processes.^92^ Poor thermal and mechanical properties limit the widespread high-value-added applications of PHAs. No biodegradable biopolymer is available globally which is thermostable. Hence, further intensive research with the novel approach is required to enhance the physical and mechanical properties of PHAs^93^ which should be thermostable and biodegradable as well without leaving any toxic effect on the environment.

The major challenges of applying PHA biopolymers are the costs of production, which are undoubtedly higher than the costs of more familiar plastics. Nonetheless, production cost of synthetic plastics is approximately $ 1250 USD per metric ton and PHA production cost vary from $ 4000 to $15000 USD per metric ton. This practice is most probably due to costly material costs and production processes, where substrates had been identified to be one of the main drivers of the costs. To cut down the expenses, it has become necessary to employ cheap feedstocks such as crop residue, food leftovers, *etc.* and generation of recombinant microbial strains. Moreover, extremophilic bacteria including Halomonas spp. can be used for the cost-effective production of PHA. However, PHAs have some problems with the low thermal and mechanical properties and more efforts are needed to improve these properties while keeping biodegradability and safe to the environment. The use of PHA biopolymers stays limited because of high production costs and inadequate manufacturing facilities and economic competition with inexpensive petrochemical plastics. The implementation of PHA biopolymers faces commercial barriers because of manufacturing technical difficulties which produce unsatisfactory material properties while making the production process short. The slow market penetration of biopolymers is due to insufficient regulatory guidelines in emerging economies and low understanding about biopolymer products among consumers. Different barriers to sustainable biopolymer adoption need technological solutions as well as government backing and educational efforts to promote sustainable plastic usage.

## 7. Conclusion and future perspectives

In the pursuit of sustainable alternatives to conventional plastics, the biosynthesis of thermostable polyhydroxyalkanoate (PHA) biopolymers from crop residues *via* bacterial processes emerges as a promising avenue. This review highlights the potential of harnessing crop residues to produce valuable biodegradable materials, addressing pressing environmental concerns linked to traditional petroleum based plastics. This approach not only offers a solution to the global plastic pollution crisis but also promotes the use of renewable and abundant agricultural resources, reducing dependence on finite fossil fuels. Moving forward, several critical areas require further exploration. Chief among them is the enhancement, identification, and engineering of bacterial strains for more efficient conversion of specific crop residues into PHA biopolymers. Process optimization is equally crucial. Through the refinement of fermentation and downstream processing techniques, overall yield and resource utilization can be maximized. Fine-tuning culture conditions, carbon source utilization, and extraction methods hold the potential to revolutionize PHA production processes. However, economic viability remains a central concern. Rigorous economic assessments are essential to ascertain the feasibility of large-scale PHA production from crop residues. Strategies aimed at minimizing production costs while maximizing benefits will be pivotal in competing with traditional plastics. A comprehensive evaluation of the environmental impact is imperative. Thorough life cycle assessments will enable us to quantify the ecological advantages of PHA production from crop residues compared to conventional plastics. Lastly, collaborative efforts among researchers, policymakers, and businesses can expedite the transition to PHA-based products and packaging materials across various industries, further advancing the shift away from petroleum-based plastics. In conclusion, the journey toward biosynthesizing thermostable PHA biopolymers from crop residues using bacterial processes has the potential to reshape the plastics landscape. With ongoing research, innovation, and collective commitment, this sustainable alternative holds the promise of revolutionizing how we approach plastic production and consumption, ushering in a greener and more responsible future. Promoting circular agriculture is imperative for effectively utilizing crop residues to mitigate emissions, boost renewable energy production, and offer economically viable alternatives that could generate additional income for farmers. Plastic pollution and crop residue burning exacerbate global warming and climate change, impeding progress towards the Sustainable Development Goals. These detrimental practices not only compromise our health and well-being (SDG 3) but also degrade air quality in urban areas (SDG 11), hamper the advancement of a circular economy (SDG 12), and directly contribute to climate change (SDG 13). Moreover, they inflict negative impacts on marine ecosystems and oceans (SDG 14).

## Data availability

The data and the materials are all available in this article and its supporting information document, which will be given access to the journal's website.

## Author contributions

Aakriti Chouhan: Conceptualization, data curation, formal analysis, and writing the original draft Preparation Professor (Dr) Archana Tiwari: Supervision, design, visualization, validation, writing – review, and editing project administration.

## Conflicts of interest

The authors declare that there is no conflict and competing interest that could have appeared to influence the paper.
